# Synthetic SARS-CoV-2 Spike-Based DNA Vaccine Elicits Robust and Long-Lasting Th1 Humoral and Cellular Immunity in Mice

**DOI:** 10.3389/fmicb.2021.727455

**Published:** 2021-09-07

**Authors:** Sawsan S. Alamri, Khalid A. Alluhaybi, Rowa Y. Alhabbab, Mohammad Basabrain, Abdullah Algaissi, Sarah Almahboub, Mohamed A. Alfaleh, Turki S. Abujamel, Wesam H. Abdulaal, M-Zaki ElAssouli, Rahaf H. Alharbi, Mazen Hassanain, Anwar M. Hashem

**Affiliations:** ^1^Vaccines and Immunotherapy Unit, King Fahd Medical Research Center, King Abdulaziz University, Jeddah, Saudi Arabia; ^2^Department of Biochemistry, Faculty of Science, King Abdulaziz University, Jeddah, Saudi Arabia; ^3^Faculty of Pharmacy, King Abdulaziz University, Jeddah, Saudi Arabia; ^4^Department of Medical Laboratory Technology, Faculty of Applied Medical Sciences, King Abdulaziz University, Jeddah, Saudi Arabia; ^5^Department of Medical Laboratories Technology, College of Applied Medical Sciences, Jazan University, Jazan, Saudi Arabia; ^6^Medical Research Center, Jazan University, Jazan, Saudi Arabia; ^7^SaudiVax Ltd., Thuwal, Saudi Arabia; ^8^Department of Surgery, Faculty of Medicine, King Saud University, Riyadh, Saudi Arabia; ^9^Department of Medical Microbiology and Parasitology, Faculty of Medicine, King Abdulaziz University, Jeddah, Saudi Arabia

**Keywords:** COVID-19, SARS-CoV-2, plasmid DNA, spike (S) glycoprotein, preclinical (*in vivo*) studies

## Abstract

The ongoing global pandemic of coronavirus disease 2019 (COVID-19) calls for an urgent development of effective and safe prophylactic and therapeutic measures. The spike (S) glycoprotein of severe acute respiratory syndrome-coronavirus (SARS-CoV-2) is a major immunogenic and protective protein and plays a crucial role in viral pathogenesis. In this study, we successfully constructed a synthetic codon-optimized DNA-based vaccine as a countermeasure against SARS-CoV-2, denoted VIU-1005. The design was based on a codon-optimized coding sequence of a consensus full-length S glycoprotein. The immunogenicity of the vaccine was tested in two mouse models (BALB/c and C57BL/6J). Th1-skewed systemic S-specific IgG antibodies and neutralizing antibodies (nAbs) were significantly induced in both models 4 weeks after three injections with 100 μg of the VIU-1005 vaccine via intramuscular needle injection but not intradermal or subcutaneous routes. Such immunization induced long-lasting IgG and memory T cell responses in mice that lasted for at least 6 months. Interestingly, using a needle-free system, we showed an enhanced immunogenicity of VIU-1005 in which lower or fewer doses were able to elicit significantly high levels of Th1-biased systemic S-specific immune responses, as demonstrated by the significant levels of binding IgG antibodies, nAbs and IFN-γ, TNF and IL-2 cytokine production from memory CD8^+^ and CD4^+^ T cells in BALB/c mice. Furthermore, compared to intradermal needle injection, which failed to induce any significant immune response, intradermal needle-free immunization elicited a robust Th1-biased humoral response similar to that observed with intramuscular immunization. Together, our results demonstrate that the synthetic VIU-1005 candidate DNA vaccine is highly immunogenic and capable of inducing long-lasting Th1-skewed humoral and cellular immunity in mice. Furthermore, we show that the use of a needle-free system could enhance the immunogenicity and minimize doses needed to induce protective immunity in mice, supporting further preclinical and clinical testing of this candidate vaccine.

## Introduction

Since its emergence in December 2019, the coronavirus disease 2019 (COVID-19) pandemic has caused ∼170 million infections with nearly more than 3.5 million deaths worldwide as of June 2021. A novel betacoronavirus (beta-CoV) known as severe acute respiratory syndrome coronavirus 2 (SARS-CoV-2) was identified as the causative agent of COVID-19 (Lu et al., 2020; Zhu et al., 2020). A zoonotic origin of SARS-CoV-2 has been suggested but has not yet been confirmed (Lu et al., 2020; Wu et al., 2020). SARS-CoV-2 can cause a wide spectrum of disease manifestations in individuals from different age groups. Most patients with COVID-19 are either asymptomatic or have mild symptoms, including fever, myalgia and cough. However, some patients can suffer from moderate to severe life-threatening acute respiratory distress syndrome (ARDS) with possible fatal outcomes (Jin et al., 2020; Rodriguez-Morales et al., 2020; Wang et al., 2020). The high human-to-human transmission rate of SARS-CoV-2 poses a major challenge toward controlling its spread (Adhikari et al., 2020; Bai et al., 2020; Xu et al., 2020b).

Coronaviruses are enveloped viruses with a positive-sense single-stranded ∼30 kb RNA genome, which encodes four structural proteins: the surface spike (S) glycoprotein, the envelope (E) protein, the membrane (M), and the nucleocapsid (N). It also encodes 16 non-structural proteins (nsp1-16) as well as putative and known accessory proteins involved in viral replication and pathogenesis (Tan et al., 2005; Fehr and Perlman, 2015; Jin et al., 2020). The viral S protein is capable of inducing a strong immune response. It is comprised of S1 and S2 subunits, in which S1 is involved in binding to the angiotensin-converting enzyme 2 (ACE2) receptor on host cells and S2 is involved in viral-host membrane fusion (Du et al., 2009; Yan et al., 2020). Therefore, neutralizing antibodies (nAbs) mainly function by targeting the receptor binding domain (RBD) in the S1 subunit and preventing viral entry in host cells. Indeed, a strong association between the magnitude of the anti-S nAb response and patient survival was recently shown in COVID-19 patients (Sun et al., 2020). Furthermore, recently approved vaccines for emergency use against SARS-CoV-2, including either mRNA- or adenovirus-based vaccines, also target the S protein of the virus. Such work as well as other studies on the Middle East respiratory syndrome coronavirus (MERS-CoV) and SARS-CoV suggest that viral S protein could represent the main target for the development of vaccines against SARS-CoV-2 (Fukushi et al., 2006, 2018; Prompetchara et al., 2020). This is further supported by the isolation and development of several therapeutic human nAbs against the SARS-CoV-2 S protein and their ability to neutralize and block viral entry and/or cell-cell spread at very low concentrations and sometimes to confer prophylactic and therapeutic protection in animals and humans (Hussen et al., 2020; Papageorgiou and Mohsin, 2020).

The ideal strategy for rapidly controlling existing and potential SARS-CoV-2 outbreaks is to develop a safe and effective vaccine. Several vaccine candidates based on full-length or truncated S protein are being developed and investigated, including DNA vaccines, RNA vaccines, replicating or non-replicating viral vectored vaccines, nanoparticle-based vaccines, whole inactivated vaccines (WIVs), and S or RBD protein-based subunit vaccines (Dong et al., 2020; Krammer, 2020; Zhang et al., 2020). Many of these vaccines are in the late stages of clinical trials and/or have been approved for emergency use in several countries. Synthetic DNA vaccines are a fast and easy platform for the production of vaccines compared to other vaccine development technologies due to their simple design, timely production, manufacturing scalability and easy and well-established quality control in addition to their temperature stability (Williams, 2013). In addition, DNA vaccines can elicit a Th1-biased immune response in contrast to other vaccine platforms, such as protein-based subunit vaccines, which could induce an undesired Th2-skewed response in the case of coronaviruses (Tseng et al., 2012; Agrawal et al., 2016).

Disposable needle-free jet injectors provide an alternative technology to conventional needle injections to deliver vaccines. They use high pressure and single-dose disposable syringes to deliver vaccines to different parts of the body including dermal, subcutaneous or muscle tissues, as required. Such technology has been proven to be immunogenic and safe in numerous clinical trials of several approved and experimental vaccines including inactivated polio vaccine (IPV), influenza vaccine, measles-mumps-rubella (MMR) vaccine, and diphtheria, tetanus, pertussis, hepatitis B, and *Haemophilus influenzae* type b conjugate (DTP-HB-Hib) pentavalent vaccine (Resik et al., 2010; Soonawala et al., 2013; McAllister et al., 2014; Bavdekar et al., 2018; Bavdekar et al., 2019; Yu and Walter, 2020). This technology was initially introduced in the 1990s and several models are approved for vaccination in the United States and Europe (Yu and Walter, 2020). Compared to traditional needle and syringe method, needle-free injectors provide several advantages including reduced cost, wastage, reuse and transport of sharps as well as lower risk of needle-stick injury and cross contamination (McAllister et al., 2014; Yu and Walter, 2020). Importantly, they provide a way for more consistent delivery of vaccines and an effective strategy for vaccine dose-sparing which could be of great value in limited vaccine supply situations or for large scale vaccination programs as we are experiencing now with COVID-19 (Resik et al., 2010; Soonawala et al., 2013; Yu and Walter, 2020).

Our previous work showed that DNA vaccines expressing MERS-CoV S1 or full-length S glycoprotein can induce Th1-skewed immune response, nAbs and S-specific T cell responses in immunized mice (Al-Amri et al., 2017). In this work, we developed a synthetic codon-optimized DNA vaccine, denoted “VIU-1005,” as a countermeasure to aid in controlling SARS-CoV-2 spread. Using a plasmid DNA vector suitable for clinical development, we describe the design and report the results from our preclinical immunogenicity testing of this vaccine candidate in two mouse models (BALB/c or C57BL/6J mice). We show that such immunization could induce significant Th1-skewed and long-lasting humoral and cellular immunity in mice. We further show that this immunogenicity could be enhanced with the use of a needle-free immunization system in which lower or fewer doses could be sufficient to elicit significant levels of systemic S-specific IgG antibodies, nAbs and T cell responses via intramuscular or intradermal immunization. Our data prove the immunogenicity of our synthetic COVID-19 DNA vaccine and support further preclinical and clinical testing of this candidate vaccine.

## Materials and Methods

### *In silico* Design of Codon-Optimized Synthetic Consensus S Protein

All available SARS-CoV-2 full-length S protein sequences (399 sequences) by March 10th, 2020 were downloaded from the GISAID database, and the dataset was filtered by removing sequences containing ambiguous amino acid codes (BJOUXZ). The final dataset was multiply aligned using Clustal Omega included in Geneious Prime 2020.1.1, the Shannon entropy for each amino acid position was determined, and the consensus protein sequence was then obtained for the full-length S glycoprotein. The coding sequence for the consensus protein sequence was then codon-optimized for human expression and synthesized by GenScript USA Inc. (Piscataway, NJ, United States).

### DNA Construct

**FIGURE 1:**
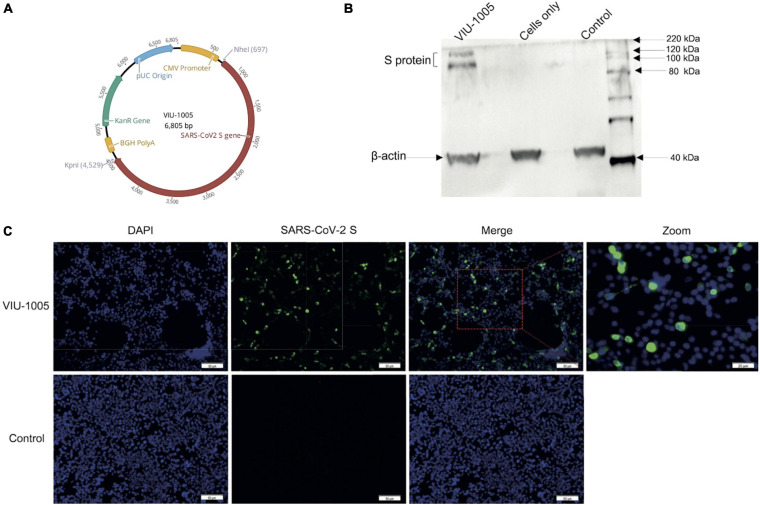
Vaccine design and expression of SARS-CoV-2 spike protein. **(A)** Schematic diagram of the SARS-CoV-2 DNA vaccine construct (VIU-1005). The SARS-CoV-2 spike gene is indicated by red color. **(B)** Western blot showing the expression of S protein at the expected size from HEK293 cells transfected with VIU-1005 construct only but not cells transfected with control plasmid or cells only control. **(C)** Immunofluorescent staining of cells transfected with VIU-1005 or control plasmid. Transfected cells were stained with anti-SARS-CoV-2 S rabbit polyclonal antibodies (green), and nuclei were counterstained with DAPI (blue). Scale bars are depicted in each image.

The designed full-length codon-optimized consensus coding sequence for the SARS-CoV-2 S protein was cloned into the mammalian expression vector pVAX1 (Invitrogen, Carlsbad, CA, United States) under the control of the cytomegalovirus immediate-early promoter and denoted VIU-1005. The coding sequence was cloned between the *Nhe*I and *Kpn*I restriction sites using the T4 DNA ligase (New England Biolabs, Ipswich, MA, United States) (Figure 1A). The construct was confirmed by restriction digestion and sequencing. Bulk endotoxin-free preparations of VIU-1005 and the empty control plasmid (control) were prepared for animal studies using the GenElute^TM^ HP Select Plasmid Gigaprep Kit (Sigma, Germany).

### Cells

The baby hamster kidney BHK-21/WI-2 cell line (Kerafast, EH1011), African green monkey kidney-derived Vero E6 cell line (ATCC, CRL-1586) and human embryonic kidney 293 (HEK293) cells (ATCC, CRL-1573) were cultured in Dulbecco’s modified essential medium (DMEM) containing penicillin (100 U/ml) and streptomycin (100 μg/ml) and supplemented with 5 or 10% fetal bovine serum (FBS) (Biosera Europe, France) in a 5% CO_2_ environment at 37°C.

### Western Blot

Briefly, 70–90% confluent HEK293 cells in 6-well plates were transiently transfected with 2 μg of VIU-1005 or control plasmid using JetPRIME^®^ Transient Transfection Protocol and Reagents (Polyplus, New York, NY, United States) according to the manufacturer’s instructions. Transfected cells were incubated at 37°C in a 5% CO_2_ incubator for 48 h. Transfected cells were then washed with phosphate-buffered saline (PBS) and lysed with radioimmunoprecipitation assay buffer (RIPA buffer) (Sigma, Germany). Proteins in the harvested cell lysates were separated using 12% SDS-PAGE, transferred to a polyvinylidene fluoride or polyvinylidene difluoride (PVDF) membrane, and subjected to western blot analysis to verify the expression of S protein using in-house rabbit anti-S (SARS-CoV-2) polyclonal antibodies, that was generated and optimized in house (Supplementary Figure 1), at a 1:2000 dilution. A horseradish peroxidase (HRP)-conjugated secondary anti-rabbit IgG antibody (Sigma, Germany), diluted 1:5000 in tris-buffered saline (TBS), was used for detection. Proteins were then detected by using iBright FL1500 (Invitrogen, Carlsbad, CA, United States).

### Immunofluorescence Analysis

HEK293 cells (70% confluent) on an 8-well cell culture slide [growth area/well (cm^2^): 0.98 and working volume/well (ml): 0.20–0.60] were transfected with 0.2 μg of VIU-1005 or control plasmid using JetPRIME^®^ Transient Transfection Protocol and Reagents (Polyplus, New York, NY, United States) according to manufacturer’s instructions, and incubated at 37°C in a 5% CO_2_ incubator for 24 h. The media was removed, and the cells were washed with PBS and fixed with 4% formaldehyde at 4°C for 10 min. Cells were then washed twice with PBS and permeabilized with PBS containing 0.2% Triton X-100 (PBS-TritonX-100) at 4°C for 20 min. Fixed cells were washed twice with PBS-TritonX-100 and the non-specific binding was blocked with 2% goat serum in PBS-TritonX-100 (blocking buffer) at room temperature for 30 min and washed twice with PBS-TritonX-100 for 5 min. Cells were then incubated with rabbit anti-SARS-CoV-2 S polyclonal antibodies in blocking buffer overnight at 1:2000 dilution at 4°C. After three washes with PBS-TritonX-100, cells were incubated with Alexa Fluor-488-labeled goat anti-rabbit IgG H&L secondary antibody (Abcam, United Kingdom) at a 1:500 dilution in blocking buffer in the dark at room temperature for 1 h. Cells were washed again three times with PBS-TritonX-100, and slides were mounted with VECTASHIELD antifade mounting medium with DAPI counterstain (Vector Laboratories, Inc., Burlingame, CA, United States). Images were captured using an Olympus BX51 fluorescence microscope and were analyzed using Image-Pro Plus software version 7.0.1 (Media Cybernetics, Inc., Rockville, MD, United States).

### Human Samples

Human samples from acutely infected patients 3–4 weeks post-onset (*n* = 10), convalescent individuals 3–4 months post-onset (*n* = 6) and individuals who received a single (*n* = 6) or two doses (*n* = 6) of BNT162b2 mRNA vaccine (3 weeks post-vaccination) were collected and used to compare the data with the immunized mouse sera. Signed informed consent forms were obtained from all participants as per institutional ethical approvals obtained from the Unit of Biomedical Ethics in King Abdulaziz University Hospital (Reference No. 245-20). All work on human samples was conducted in accordance with the Declaration of Helsinki.

### Animal Studies

Six- to 8-week-old female BALB/c or C57BL/6J mice were obtained from the animal facility in King Fahd Medical Research Center (KFMRC), King Abdulaziz University (KAU), Jeddah, Saudi Arabia. All animal experiments were conducted in accordance with the guidelines and approval of the Animal Care and Use Committee (ACUC) at KFMRC and ethical approval from the bioethical committee at KAU (approval number 04-CEGMR-Bioeth-2020). The study is reported in accordance with ARRIVE guidelines. In one experiment, two groups of BALB/c or C57BL/6J mice (10 per group) were intramuscularly immunized via needle injection with 3 doses of 100 μg of either VIU-1005 or control plasmid at 2-week intervals, and blood samples were collected for serological testing every 2 weeks starting from day 0 (prebleed) until week 8. In another experiment, three groups of BALB/c mice (5 per group) were immunized intramuscularly, intradermally or subcutaneously via needle injection with 3 doses of 100 μg of either VIU-1005 or control plasmid at 2-week intervals, and blood samples were collected at different time points until week 25 post primary immunization. In a third experiment, BALB/c mice (4–5 per group) were intramuscularly or intradermally immunized with 3 doses of 25, 50, or 100 μg of VIU-1005 plasmid at 2-week intervals using either conventional needle injection or customized needle-free Tropis system (PharmaJet, Golden, CO, United States) suitable for use in small animals to deliver vaccines intradermally, subcutaneously or intramuscularly, and blood samples were collected every week until week 8 post primary immunization. Spleens were collected from some animals for flow cytometry analysis as outlined below.

### Indirect ELISA

The end-point titers or optical density (OD) readings at a 1:100 dilution of total anti-S1 IgG or its isotypes (IgG1, IgG2a, and IgG2b) from immunized mice were determined by enzyme-linked immunosorbent assay (ELISA) as described previously (Al-Amri et al., 2017). Briefly, 96-well EU Immulon 2 HB plates (Thermo Scientific) were coated overnight at 4°C with the SARS-CoV-2 S1 subunit (amino acids 1–685) (Sino Biological, China) at 1 μg/ml in PBS (50 μl/well). Then, the plates were washed three times with washing buffer [PBS containing 0.1% Tween-20 (PBS-T)]. This was followed by blocking with 200 μl/well of blocking buffer (5% skim milk in PBS-T) for 1 h at room temperature. Plates were washed three times and incubated with a 2-fold serial dilution of mouse sera (100 μl/well) starting from a 1:100 dilution in blocking buffer and incubated for 1 h at 37°C. Some samples collected at different time points were only tested at a 1:100 dilution. After three washes, peroxidase-conjugated rabbit anti-mouse IgG secondary antibodies as well as anti-IgG1, IgG2a, or IgG2b antibodies (Jackson Immunoresearch Laboratories, West Grove, PA, United States) were added at dilutions recommended by the manufacturer and incubated for 1 h at 37°C at 100 μl/well. Anti-IgG2a antibody used is cross-reactive to IgG2c and thus it was used to detect antibodies in sera collected from BALB/c and C57BL/6J mice; we refer to both IgG2a and IgG2c as IgG2a. Excess secondary antibodies were removed by three washes, and color was developed by adding 3,3′,5,5′-tetramethylbenzidine (TMB) substrate (KPL, Gaithersburg, MD, United States) for 15 min. Finally, reactions were stopped with 0.16 M sulfuric acid, and the absorbance was read spectrophotometrically at 450 nm using a Synergy 2 Multi-Detection Microplate Reader (BioTek, Winooski, VT, United States). End-point titers were determined and expressed as the reciprocals of the highest dilution with OD reading above the cut-off value defined as the mean of the control group plus three standard deviations (SD).

### SARS-CoV-2 Pseudovirus Neutralization Assay

The pseudovirus microneutralization assay was performed as previously described (Almahboub et al., 2020). Briefly, rVSV-ΔG/SARS-2-S^∗^-luciferase pseudovirus was generated by transfecting BHK21/WI-2 cells with pcDNA expressing codon-optimized full-length SARS-CoV-2 S protein (GenBank accession number: MN908947) using Lipofectamine^TM^ 2000 transfection reagent (Invitrogen, Carlsbad, CA, United States). Transfected cells were then infected with rVSV-ΔG/G^∗^-luciferase 24 h later, and the supernatant containing the generated rVSV-ΔG/SARS-2-S^∗^-luciferase pseudovirus was collected 24 h post-infection. The collected virus was titrated by measuring luciferase activity from serially diluted supernatant on Vero E6 cells, and the titer was expressed as a relative luciferase unit (RLU). A neutralization assay was then conducted by incubating two-fold serial dilutions of heat-inactivated mouse sera from vaccinated and control groups starting from a 1:20 dilution (in duplicate) with DMEM-5% FBS containing 5 × 10^4^ RLU rVSV-ΔG/SARS-2-S^∗^-luciferase pseudovirus for 1 h at 37°C in a 5% CO_2_ incubator. Pseudovirus–serum mixtures were transferred onto confluent Vero E6 cell monolayers in white 96-well plates and incubated for 24 h at 37°C in a 5% CO_2_ incubator. After 24 h, cells were lysed, luciferase activity was measured using a Luciferase Assay System (Promega) according to the manufacturer’s instructions, and luminescence was measured using a BioTek Synergy 2 microplate reader (BioTek, Winooski, VT, United States). Cell-only control (CC) and virus control (VC) were included with each assay run. The median inhibitory concentration (IC_50_) of neutralizing antibodies (nAbs) was determined using a four-parameter logistic (4PL) curve in GraphPad Prism V8 software (GraphPad Co.) and calculated as the reciprocal of the serum dilution at which RLU was reduced by 50% compared with the virus control wells after subtraction of the background RLUs in the control groups with cells only.

### Flow Cytometry

The expression of interferon (IFN-γ), tumor necrosis factor–α (TNF-α) and interleukin-2 (IL-2) responses from memory CD8^+^ and CD4^+^ T cells was evaluated at different time points after the last immunizations from immunized animals. Single-cell suspensions of splenocytes were prepared from individual immunized and control BALB/c mice. In brief, spleens from mice were collected in 3 ml of RPMI 1640 (Invitrogen, Carlsbad, CA) supplemented with 5% heat inactivated FBS and smashed between frosted ends of two glass slides. Processed splenocytes were then filtered through 70-μm nylon filters and centrifuged at 430 × *g* for 5 min at room temperature. Red blood cells were then lysed by adding 3 ml of ammonium chloride potassium (ACK) lysis buffer (Invitrogen, Carlsbad, CA, United States) for 4 min at room temperature, and an equal volume of PBS was then added for neutralization. Cells were centrifuged again at 430 × *g* for 5 min at room temperature, and cell pellets were resuspended in RPMI 1640 at a concentration of 1 × 10^7^ cells/ml. One million cells per well were added to a U-bottom 96-well plate and stimulated with 5 μg/ml pools of 15-mer peptides overlapping by 11 residues and covering the entire S protein (GenScript USA Inc., Piscataway, NJ, United States). Stimulation was performed by incubation for 6 h at 37°C and 5% CO_2_ in the presence of Protein Transport Inhibitor Cocktail (brefeldin A) (BD Biosciences, San Jose, CA, United States) at a dilution of 1:1000. Splenocytes stimulated in the presence of phorbol 12-myristate 13-acetate (PMA) and ionomycin as a positive control, and in RPMI 1640 medium as a negative control. Cells were then washed in FACS buffer (PBS with 2% heat inactivated FBS) and stained with a LIVE/DEAD^TM^ Fixable Near-IR Dead Cell Stain Kit, for 633 or 635 nm excitation (Invitrogen, Carlsbad, CA, United States) for 30 min at room temperature. After washing with PBS, Pacific Blue-conjugated anti-mouse CD8, Pacific Blue-conjugated anti-mouse CD4, APC-conjugated anti-mouse CD44 and Pe-Cy7-conjugated anti-mouse CD62L antibodies (BioLegend, United Kingdom) were used for surface marker staining. CD8 and CD4 cells were evaluated in separate experiments. The cells were then washed with FACS buffer, fixed and permeabilized using Cytofix/Cytoperm Solution (BD Biosciences, San Jose, CA) according to the manufacturer’s protocol. For intracellular staining, cells were labeled with anti-mouse FITC-conjugated anti-IFN-γ (clone XMG1.2), PE-conjugated anti-TNF-α (clone MP6-XT22) and PerCP/Cy5.5–conjugated anti-IL-2 (clone JES6-5H4) antibodies (BioLegend, United Kingdom) for 20 min at 4°C. Cells were then washed twice with permeabilization buffer and once with FACS buffer. All data were collected using BD FACSAria^TM^ III flow cytometer (BD Biosciences, San Jose, CA, United States) and analyzed using FlowJo v10 software (Tree Star).

### Statistical Analysis

Statistical analyses and graphical presentations were conducted using GraphPad Prism version 8.0 software (GraphPad Software, Inc., La Jolla, CA, United States). Statistical analysis was conducted using one-way analysis of variance with the Bonferroni *post hoc* test to adjust for multiple comparisons between groups or the Mann–Whitney test. All values are depicted as the mean ± SD, and statistical significance is reported as ^∗^*P* ≤ 0.05, ^∗∗^*P* ≤ 0.01, ***p ≤ 0.001, and ****p ≤ 0.0001.

## Results

### *In vitro* Protein Expression From the Synthetic DNA Vaccine

Prior to animal experiments, spike protein expression from the VIU-1005 construct (Figure 1A) was confirmed *in vitro* in HEK293 cells. Western blot analysis confirmed that the recombinant construct was able to express the spike protein indicated by bands observed at the expected molecular weights but not in lysates from cells only or cells transfected with the control plasmid, as shown in Figure 1B. Immunofluorescence analysis was also performed to visualize the expression of the SARS-CoV-2 spike protein in transfected HEK293 cells. As shown in Figure 1C, strong S protein expression was only detected in cells transfected with VIU-1005 using rabbit anti-SARS-CoV-2 S polyclonal antibodies but not in cells transfected with the control plasmid. The detection of the S protein expressed from VIU-1005 by polyclonal anti-S antibodies suggests that the protein maintained its structural confirmation.

### Conventional Needle Intramuscular Immunization With the VIU-1005 Vaccine Elicits a Significant Th1-Skewed Humoral Immune Response and nAbs in Both BALB/c and C57BL/6J Mouse Models

**FIGURE 2:**
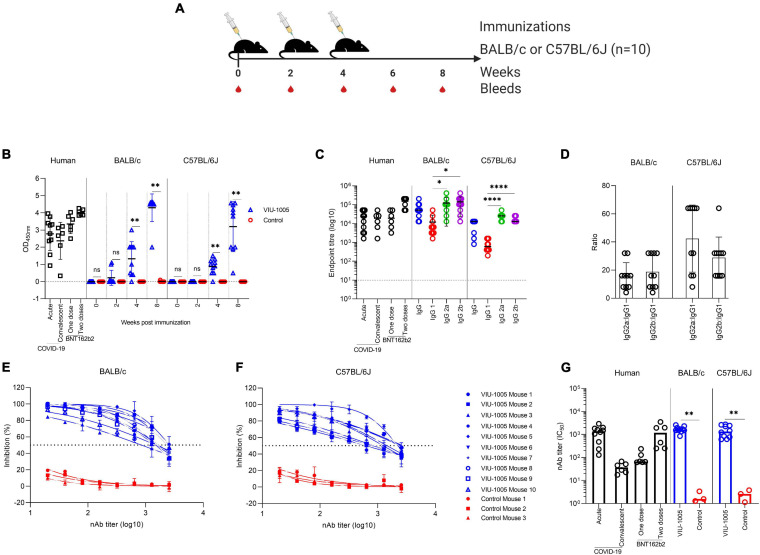
Antibody response against SARS-CoV-2 after intramuscular needle-based immunization in BALB/c and C57BL/6J mice. **(A)** Mice (*n* = 10) were intramuscularly immunized via needle injection with 3 doses of 100 μg at 2-week intervals using either VIU-1005 or control plasmid. The timeline shows the bleeding and immunization regimen. **(B)** OD values of S1-specific binding total IgG at a 1:100 dilution from each mouse were determined by ELISA at 2, 4, and 8 weeks post first immunization on day 0. IgG responses from acutely infected patients (3–4 weeks post-onset), convalescent individuals (3–4 months post-onset) and individuals who received a single or two doses of BNT162b2 mRNA vaccine (3 weeks post-vaccination) are also shown to compare the data with the immunized mouse sera. **(C)** End-point titers of S1-specific total IgG, IgG1, IgG2a, and IgG2b were determined by ELISA in samples collected at week 8 from immunized mice. End-point titers of total IgG from acutely infected patients (3–4 weeks post-onset), convalescent individuals (3–4 months post-onset) and individuals who received a single or two doses of BNT162b2 mRNA vaccine (3 weeks post-vaccination) are also shown to compare the data with the immunized mouse sera. **(D)** IgG2a:IgG1 and IgG2b:IgG1 ratios were calculated in samples collected from immunized mice at week 8. Serum samples from **(E)** BALB/c or **(F)** C57BL/6J mice that were intramuscularly immunized with 3 doses of 100 μg at 2-week intervals using either VIU-1005 (*n* = 10) or control plasmid (*n* = 3) were collected at week 8 post first immunization and serially diluted and tested in duplicate for their neutralizing activity against rVSV-ΔG/SARS-2-S*-luciferase pseudovirus as described in the “Materials and Methods.” **(E,F)** Neutralizing activity from serum samples collected from each mouse and **(G)** nAb titers (IC_50_). nAbs from acutely infected patients (3–4 weeks post-onset), convalescent individuals (3–4 months post-onset) and individuals who received a single or two doses of BNT162b2 mRNA vaccine (3 weeks post-vaccination) were used as a control for the assay and to compare the data with the immunized mouse sera. Data are shown as the mean ± SD for each group from one experiment in **(B–F)**, and bars represent the mean in **(G)**. Statistical significance was determined by the Mann–Whitney test in **(B,G)** and one-way analysis of variance with the Bonferroni *post hoc* test in **(C)**.

Next, we investigated the immunogenicity induced by the generated synthetic DNA vaccine candidate (VIU-1005) in BALB/c and C57BL/6J mice (Figure 2A). The two mouse models were used to evaluate the type and nature of the immune response, as BALB/c and C57BL/6J mice phenotypically represent Th2- and Th1-skewed models, respectively (Kuroda et al., 2000; Fornefett et al., 2018). Mice intramuscularly immunized with three doses of the vaccine induced significant levels of S1-specific IgG. Specifically, while we observed that immunization with two doses elicited significant levels of S1-specific IgG in both BALB/c and C57BL/6J mice (i.e., on week 4), samples collected 4 weeks post-third immunization (i.e., on week 8) showed higher and more significant levels compared to the control groups immunized with the control plasmid (Figure 2B). Interestingly, immunization with VIU-1005 significantly induced higher levels of S1-specific IgG2a and IgG2b isotypes compared to IgG1 in both animal models (Figure 2C). As expected, the bias toward Th1 response was more pronounced in C57BL/6J mice since they are Th1-prone animals compared to BALB/c mice, as shown by the high IgG2a/IgG1 or IgG2b/IgG1 ratios (Figure 2D). Nonetheless, BALB/c mice also showed a Th1-skewed response, confirming that VIU-1005 could elicit elevated titers of S1-specific IgG2a and IgG2b antibodies, which is indicative of a skewed Th1 immune response despite the genetic and phenotypic background of the animal model. In all cases, three doses of VIU-1005 elicited levels of binding antibody equivalent or higher to those observed in acutely infected or recovered patients as well as the levels observed in people immunized with BNT162b2 mRNA vaccine. However, one should consider the differences in secondary antibodies used for human and mouse samples and their possible effect on the level of the generated binding signals.

To further investigate the effector function of the elicited antibodies generated by the developed vaccine, sera from immunized and control mice were tested in pseudovirus microneutralization assay to evaluate nAbs. As shown in Figures 2E–G, sera collected at week 8 from the VIU-1005-immunized group induced significant levels of nAbs compared to the control group, with mean IC_50_ titers of 1 × 10^3^ in both BALB/c and C57BL/6J mice (Figure 2G). As expected, no neutralizing activity was observed in mice immunized with the control plasmid. Importantly, nAb levels observed in both mouse models were equivalent to the levels found in acutely infected COVID-19 patients and individuals vaccinated with two doses of BNT162b2 mRNA vaccine but not in convalescent people or those who received a single dose of the BNT162b2 vaccine.

### Intramuscular Immunization With the VIU-1005 Vaccine Elicits Long-Lasting Humoral and Cellular Immune Responses in Mice

**FIGURE 3:**
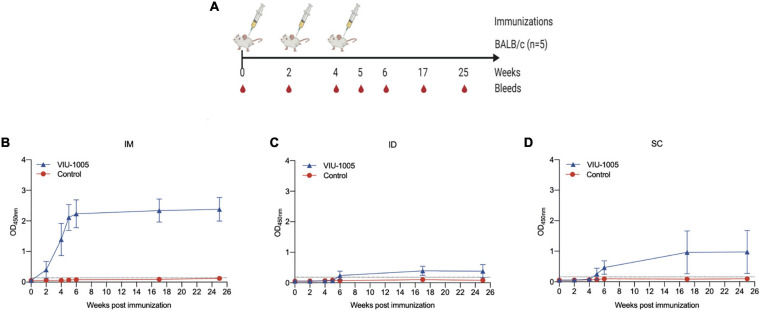
Long-term IgG response in mice immunized with different routes. **(A)** BALB/c mice were immunized via different routes using 3 doses (100 μg each) at 2-week intervals using either VIU-1005 or control plasmid. The timeline shows the bleeding and immunization regimen. OD values of S1-specific binding total IgG at a 1:100 dilution from each mouse immunized via **(B)** intramuscular, **(C)** intradermal, or **(D)** subcutaneous routes were determined by ELISA at 2, 4, 5, 6, 17, and 25 weeks post first immunization on day 0. Data are shown as the mean ± SD for each group from one experiment (*n* = 5).

**FIGURE 4:**
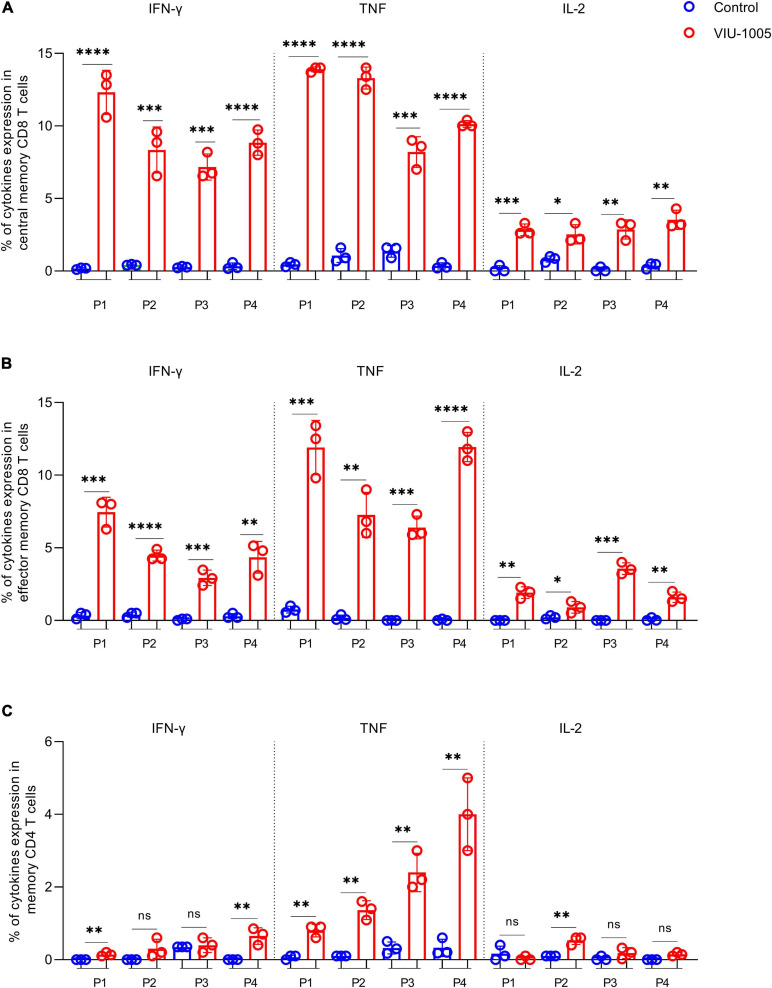
Long-lasting cellular immune response against SARS-CoV-2 S protein in BALB/c mice. Intramuscularly immunized BALB/c mice with 100 μg of VIU-1005 or control plasmid using needle injection were sacrificed at 25 weeks after the primary immunization, and splenocytes (*n* = 3) were isolated and restimulated *ex vivo* with synthetic peptide pools covering SARS-CoV-2 S protein. Gating strategy on live CD8^+^CD44^+^CD62L^+^ central memory T cells (CD8^+^ TCM), effector CD8^+^CD44^+^CD62L^–^ memory T cells (CD8^+^ TEM), and memory CD4^+^CD44^+^CD62L^–^ T cells, and representative plots are shown in Supplementary Figure 2. Histograms display IFN-γ, TNF-α, and IL-2 expression on stimulated CD8^+^ TCM, CD8^+^ TEM, and memory CD4^+^ T populations from immunized BALB/c mice after restimulation with the different peptide pools. Data are shown as the mean ± SD for each group from one experiment (*n* = 3).

To determine whether the route of immunization can affect the immunogenicity of our vaccine candidate and to measure the longevity of the generated antibody response in mice, we immunized BALB/c mice with different immunization routes and compared the immunogenicity of VIU-1005 over an extended period of time (Figure 3A). Interestingly, intradermal and subcutaneous immunization of BALB/c mice with three doses of VIU-1005 failed to induce any significant IgG levels (Figures 3C,D). On the other hand, such immunization via the intramuscular route elicited long-lasting S1-specific IgG that lasted until week 25 post-primary immunization (Figure 3B). Having observed the Th1-skewed response in VIU-1005-immunized mice, we further tried to evaluate S-specific memory CD8^+^ and CD4^+^ T cell responses. To this end, splenocytes isolated from BALB/c mice immunized with 100 μg of VIU-1005 using needle injection were restimulated *ex vivo* with four SARS-CoV-2 peptide pools covering the SARS-CoV-2 S protein. Flow cytometric analysis showed that antigen-specific CD8^+^CD44^+^CD62L^+^ central memory T cells (CD8^+^ TCM), effector CD8^+^CD44^+^CD62L^–^ memory T cells (CD8^+^ TEM), and memory CD4^+^CD44^+^CD62L^–^ T cells were maintained until week 25 post primary immunization in VIU-1005-immunized animals only compared to the group immunized with the control plasmid. Specifically, *ex vivo* restimulation of splenocytes from mice immunized with peptide pools resulted in significantly high levels of IFN-γ, TNF-α and IL-2 in CD8^+^ TCM and TEM cells (Figure 4). On the other hand, only TNF-α was significantly produced by memory CD4^+^ T cells. Gating strategy and representative FACS plots are shown in Supplementary Figure 2. Taken together, these data clearly show that intramuscular immunization with this synthetic codon-optimized DNA vaccine could induce long-lasting antibody and T cell responses in mice.

### Needle-Free Intramuscular Immunization Enhances the Immunogenicity of the VIU-1005 Vaccine in Mice

**FIGURE 5:**
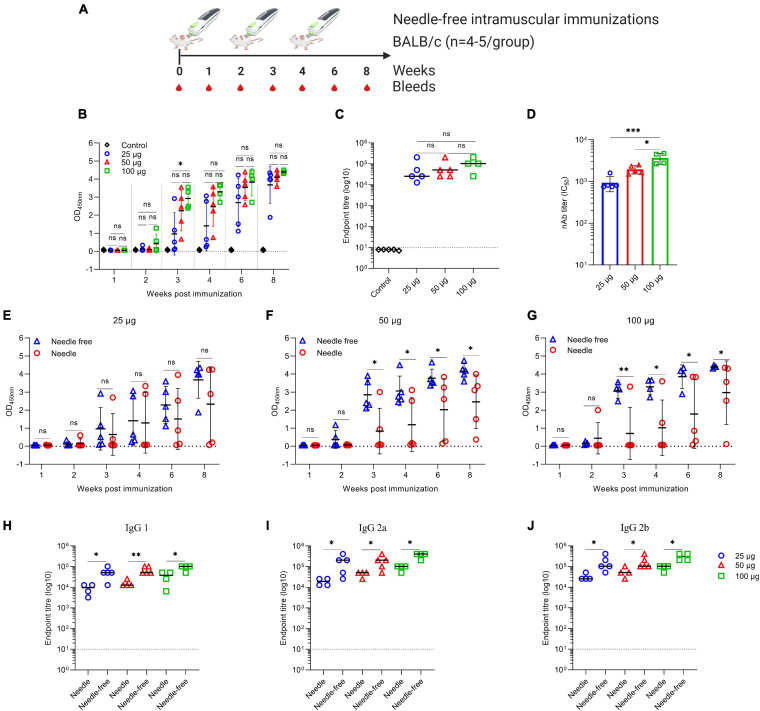
Antigen-specific antibody response in BALB/c mice intramuscularly immunized with VIU-1005 using either needle-based or needle-free immunization. **(A)** Timeline of bleeding and immunization regimen. BALB/c mice were immunized intramuscularly with 3 doses of 25, 50, or 100 μg at 2-week intervals using the VIU-1005 vaccine or control plasmid. **(B)** OD values of S1-specific binding total IgG at a 1:100 dilution from each mouse were determined by ELISA at 1, 2, 3, 4, 6, and 8 weeks post first immunization on day 0. **(C)** The end-point titer of S1-specific total IgG was determined by ELISA in samples collected from immunized mice at week 8. **(D)** nAb titers (IC_50_) from mice immunized with doses of 25, 50, or 100 μg of VIU-1005 plasmid using a needle-free system were determined at week 8. **(E–G)** OD values of binding total S1-specific IgG at a 1:100 dilution from 1, 2, 3, 4, 6, and 8 weeks post-first immunization on day 0 in groups immunized with 3 doses of **(E)** 25 μg, **(F)** 50 μg, or **(G)** 100 μg of VIU-1005 plasmid at 2-week intervals using either needle injection or a needle-free Tropis system. **(H–J)** End-point titers of S1-specific **(H)** IgG1, **(I)** IgG2a, and **(J)** IgG2b were determined by ELISA in samples collected on week 8 from immunized mice. Data are shown as the mean ± SD from one experiment (*n* = 4–5). Statistical significance was determined by one-way analysis of variance with Bonferroni *post hoc* test in **(B–D)** and Mann–Whitney test in **(E–J)**.

To further improve the immunogenicity of the naked synthetic DNA vaccine and minimize the number and size of doses, we investigated the use of a needle-free Tropis system with our vaccine in BALB/c mice in a separate experiment (Figure 5A). Needle-free immunization with only two or three doses of as low as 25 μg of the vaccine via the intramuscular route was able to elicit significantly high levels of S1-specific total IgG. Specifically, two or three doses of VIU-1005 were able to induce high levels of S1-specific total IgG in a dose-dependent fashion (Figure 5B), in which two doses of 50 and 100 μg administered by the needle-free system elicited S1-specific total IgG levels that were equivalent to or higher than those induced by three doses of 100 μg by needle injection, as shown in Figure 2B. High endpoint titers of S1-specific total IgG from week 8 sera of mice intramuscularly immunized with three doses of 25, 50, and 100 μg of VIU-1005 were also observed (Figure 5C). As expected, no S1-specific IgG antibody response was observed in mice immunized with the control plasmid. While we observed a dose-dependent immune response in immunized animals, no significant differences were observed for S1-specific total IgG in the sera of mice immunized with 25, 50, and 100 μg of VIU-1005 from week 4 onward.

We further investigated the neutralizing activity of the sera from BALB/c mice immunized with the different doses of VIU-1005 vaccine using a needle-free Tropis system. As shown in Figure 5D, the results demonstrated that all three tested doses were able to induce potent titers of nAbs in a dose-dependent manner against SARS-CoV-2 pseudovirus in Vero cells. Immunization with three doses of as low as 25 μg of the vaccine via the intramuscular route administered by the needle-free system was sufficient to induce high levels of nAbs, reaching 1 × 10^3^, which are equivalent to those induced by three doses of 100 μg by needle injection (Figure 2G). On the other hand, 50 and 100 μg of VIU-1005 elicited higher levels of nAbs, with means of ∼2 × 10^3^ and ∼3 × 10^3^, respectively. These results suggest that a dose of 25 or 50 μg of VIU-1005 is sufficient to induce high titers of S1-specific antibodies and nAbs in mice using the needle-free system.

To further confirm the superiority of the needle-free system compared to conventional needle injection, we compared S1-specific total IgG responses in sera collected from BALB/c mice immunized with 25 μg (Figure 5E), 50 μg (Figure 5F), and 100 μg (Figure 5G) using both immunization methods. As shown in Figure 5, needle-free administration of 50 and 100 μg not only induced significantly high levels of S1-specific total IgG compared to needle injection, but we also observed that all mice immunized with the needle-free system elicited S1-specific IgG compared to a few from the group immunized using needle injection. Of note, variation in the induced levels of binding antibodies by conventional needle injection was high compared to mice immunized using the needle-free system (Figures 2B, 5E–G). Furthermore, needle-free immunization with VIU-1005 significantly induced higher levels of S1-specific IgG1, IgG2a, and IgG2b isotypes than conventional needle injection in BALB/c mice, with elevated titers of IgG2a and IgG2b antibodies, suggesting a bias toward the Th1 response at the three tested doses (Figures 5H–J).

**FIGURE 6:**
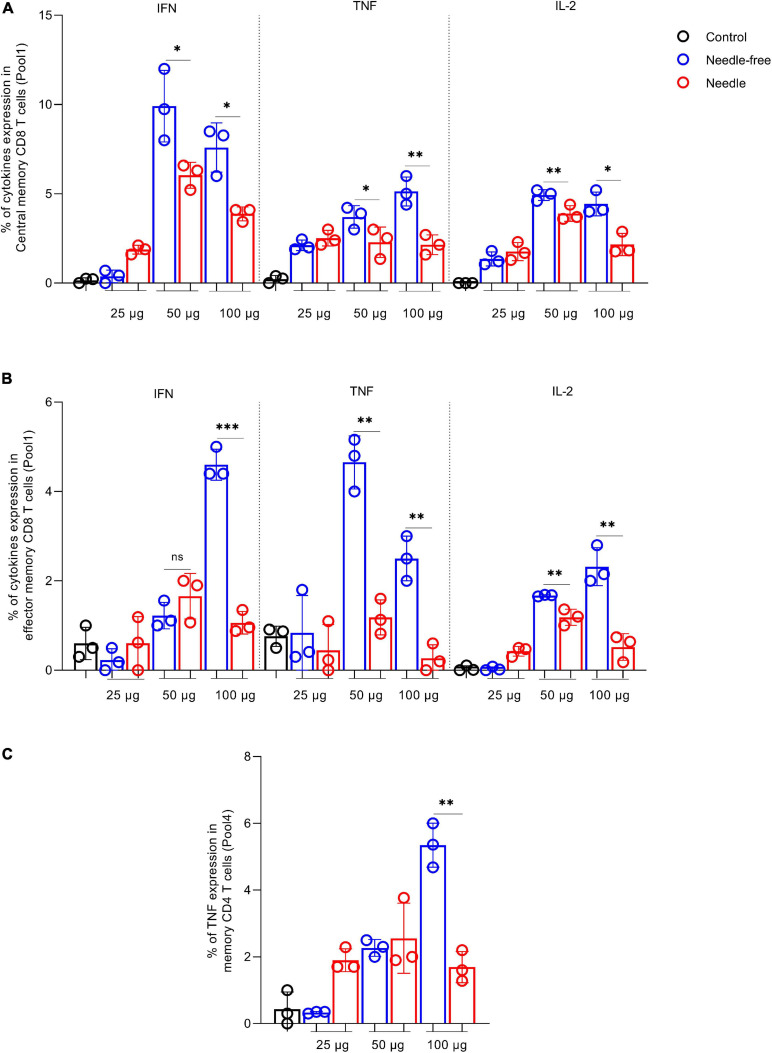
Memory T cell response against SARS-CoV-2 S protein in BALB/c mice intramuscularly immunized with VIU-1005 using either needle-based or needle-free immunization. Intramuscularly immunized BALB/c mice with 3 doses of 25, 50, or 100 μg of VIU-1005 plasmid at 2-week intervals using either needle injection or needle-free Tropis system were sacrificed at 12 weeks after the primary immunization, and splenocytes (*n* = 3) were isolated and restimulated *ex vivo* with synthetic peptide pool 1 from SARS-CoV-2 S protein. Representative plots are shown in Supplementary Figure 3. Histograms show IFN-γ, TNF-α and IL-2 on **(A)** live CD8^+^CD44^+^CD62L^+^ central memory T cells (CD8^+^ TCM) and **(B)** effector CD8^+^CD44^+^CD62L^–^ memory T cells (CD8^+^ TEM). **(C)** TNF-α expression in memory CD4^+^CD44^+^CD62L^–^ T cells. Data are shown as the mean ± SD for each group from one experiment (*n* = 3).

We also investigated the memory response from CD8^+^ and CD4^+^ T cells 12 weeks post primary immunization using peptide pool 1, which resulted in the highest memory response upon *ex vivo* restimulation, especially in CD8^+^ T cells. As shown in Figure 6, CD8^+^ TCM (Figure 6A) and CD8^+^ TEM (Figure 6B) produced significant levels of IFN-γ, TNF-α, and IL-2. Additionally, memory CD4^+^ T cells (Figure 6C) showed high levels of TNF-α. Remarkably, 50 and 100 μg of the VIU-1005 vaccine resulted in significantly higher induction of IFN-γ, TNF-α, and IL-2 when administered via needle-free immunization compared to conventional needle injection except for IFN-γ at the 50 μg dose (Figures 6A,B). A similar finding was observed for TNF-α in memory CD4^+^ T cells when 100 μg of VIU-1005 was used (Figure 6C). Representative FACS plots are shown in Supplementary Figure 3. Together, these results showed that needle-free immunization not only enhanced S1-specific IgG levels but also boosted Th1 responses, as demonstrated by markedly elevated titers of S1-specific IgG2a and IgG2b antibodies and memory responses from CD8^+^ and CD4^+^ T cells.

### Needle-Free Intradermal Immunization With the VIU-1005 Vaccine Induces a Robust S1-Specific Humoral Response in Mice

**FIGURE 7:**
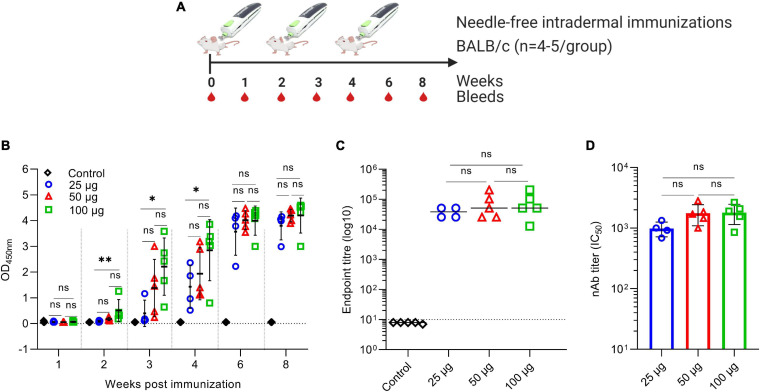
Antibody response against SARS-CoV-2 in BALB/c mice intradermally immunized with VIU-1005 using a needle-free Tropis system. **(A)** Timeline of bleeding and immunization regimen. BALB/c mice were immunized intradermally with 3 doses of 25, 50, or 100 μg at 2-week intervals using the VIU-1005 vaccine or control plasmid. **(B)** OD values of S1-specific binding total IgG at a 1:100 dilution from each mouse were determined by ELISA at 1, 2, 3, 4, 6, and 8 weeks post first immunization on day 0. **(C)** The end-point titer of S1-specific total IgG was determined by ELISA in samples collected from immunized mice at week 8. **(D)** nAb titers (IC_50_) from mice immunized with doses of 25, 50, or 100 μg of VIU-1005 plasmid using a needle-free system were determined at week 8. Data are shown as the mean ± SD from one experiment (*n* = 4–5). Statistical significance was determined by one-way analysis of variance with Bonferroni *post hoc* test.

Interestingly, while intradermal and subcutaneous needle injection using three doses of 100 μg failed to induce high or significant levels of specific IgG (Figures 3C,D), two or three doses of VIU-1005 administered intradermally via the needle-free system (Figure 7A) induced significantly high levels of S1-specific IgG in a dose-dependent fashion (Figure 7B). Importantly, the levels of S1-specific IgG were equivalent to those generated by three doses administered by intramuscular needle injection. When endpoint IgG titers from the week 8 sera of mice immunized with the three doses of 25, 50, and 100 μg were compared, we observed an induction of high levels of IgG antibody response in mice immunized intradermally with 25 μg of VIU-1005 to levels similar to those observed in mice immunized with higher doses with no significant differences (Figure 7C). Importantly, such immunization also elicited high titers of nAbs with an IC_50_ of more than 1 × 10^3^, especially with 50 and 100 μg doses (Figure 7D). These data clearly show that intradermal needle-free immunization, even at a low dose, could elicit potent humoral immunity in mice, further confirming the superiority of the needle-free system compared to conventional needle injection.

## Discussion

There is an urgent need to develop a safe and protective vaccine against SARS-CoV-2 to help control the COVID-19 pandemic. Synthetic DNA vaccines represent a promising platform to use in response to outbreaks such as COVID-19. They could be quickly designed and synthesized based on viral sequences. Their manufacturing is easy and scalable, unlike other platforms, such as viral vectors or virus-based vaccines (Xu et al., 2014; Silveira et al., 2017). They are very stable under different storage conditions, which can be extremely practical for use in rural regions (Ingolotti et al., 2010). While the use of consensus S could induce a broad immune response by using conserved sequences that should cover some possible variation in viral sequences, some variants could emerge and show reduced susceptibility to antibodies induced by some vaccines, as we recently observed with some variants of concern such as the beta (B.1.351; 20H/501Y.V2), gamma (P.1; 20J/501Y.V3), and delta (B.1.617.2; 21A/S:478K) variants emerged in South Africa, Brazil and India, respectively. Nonetheless, synthetic DNA vaccines can be quickly adapted and deployed to countermeasure such variants of concern.

Numerous COVID-19 vaccines are in different stages of the developments and some have already gained emergency approval. Among them, there are at least 7 candidate vaccines based on plasmid DNA technology in clinical trials. The most advanced DNA vaccine against COVID-19 is Inovio Pharmaceuticals DNA Vaccine (INO-4800) which has demonstrated a safe profile in volunteers as well as strong humoral and cellular immune responses after two intradermal injections of either 1 or 2 mg dose followed by electroporation (Smith et al., 2020; Mammen et al., 2021). INO-4800 is currently being evaluated in a large phase 3 trials at several locations around the world.

Based on our previous work in developing a vaccine against MERS-CoV as well as other vaccines developed for other coronaviruses, we selected a consensus sequence of SARS-CoV-2 S protein as of March 10th, 2020 as a target because it is a major protein on the surface of the virus and a main target for nAbs. *In vivo* testing in mice showed that intramuscular immunization with three doses of VIU-1005 via needle injection induced significant and long-lasting levels of Th1-skewed S1-specific immune responses in BALB/c (Th2-dominant mouse strain) and C57BL/6J (Th1-dominant mouse strain) mice (Kuroda et al., 2000; Fornefett et al., 2018). The vaccine also elicited significant levels of nAbs comparable to those observed in acutely infected patients and individuals vaccinated with the BNT162b2 mRNA vaccine, with mean IC_50_ titers of 1 × 10^3^ in both models. Importantly, our findings show that this vaccine could induce long-lasting humoral and cellular immunity that was detected for at least 25 weeks after primary immunization.

Notably, needle-free immunization with doses as low as 25 μg of VIU-1005 via either intramuscular or intradermal routes was able to elicit high levels of S1-specific IgG in a dose-dependent fashion in BALB/c mice. Two or three doses of 50 and 100 μg administered by the needle-free system elicited IgG and nAb levels that were equivalent to or higher than those induced by three doses of 100 μg by needle injection in BALB/c mice. Interestingly, using a needle-free system enhanced the immunogenicity of the VIU-1005 vaccine and induced significant levels of S1-specific IgG when given intradermally, albeit the inability of the vaccine to induce any levels of antibodies when administered intradermally via needle injection. Furthermore, the use of needle-free immunization enhances the induction of memory T cell immunity, as demonstrated by the increase in IFN-γ, TNF-α, and IL-2 cytokine production from both memory CD4^+^ and CD8^+^ T cell compartments, which is along with significantly elevated titers of IgG2a and IgG2b, suggesting successful induction of Th1-skewed immune responses, as recently reported. Nonetheless, while measurement of Th2 cytokine expression from spike-reactive T cells such as IL-4, IL-5, and IL-13 would have provided more conclusively prove of the Th1/Th2 bias of the immune response, it is well documented in literature that bias in IgG subclasses could be used as a surrogate to identify such changes as they reflect the nature of cytokines being produced in the microenvironment.

Unlike other leading DNA vaccine against SARS-CoV-2, the use of the needle-free system can not only lead to the induction of a higher and consistent immune response but also has other advantages, including a lower amount and less doses of vaccines and higher antigen expression. Clinically, the needle-free system is associated with less pain and stress than the conventional needle system. As pian at the site of injection was one of the most common adverse events of the currently approved SARS-CoV-2 vaccines, a needle-free system could represent an ideal alternative to administer vaccines (Resik et al., 2010; Soonawala et al., 2013; Yu and Walter, 2020). Furthermore, similar to other methods such as electroporation and microneedle delivery, needle-free jet injection represents an alternative physical delivery mechanism to enhance vaccines immunogenicity. Through precise parenteral administration of vaccines under high pressure, needle-free injectors effectively disperse and deposit vaccines at the desired tissue which results in consistent delivery and improved uptake of vaccines.

Nonetheless, while we show the usefulness of the needle-free system in enhancing immunogenicity of our DNA vaccine, it is important to highlight that other methods such as electroporation has also been shown to enhance immunogenicity of several DNA vaccines including COVID-19 INO-4800, MERS-CoV vaccine (INO-4700), Zika vaccine (GLS-5700), and others (Tebas et al., 2017; Jiang et al., 2019; Smith et al., 2020; Xu et al., 2020a; Mammen et al., 2021). Furthermore, head-to-head comparison of needle-free systems with electroporation has shown that electroporation was better in enhancing immunogenicity of DNA vaccine compared to Biojector^®^ B2000 needle-free delivery device which is different than the one used in our study. Interestingly, it was found that combining both methods could enhance immunogenicity even further in different animal models, suggesting that further studies investigating combined method should be pursued.

The fact that this vaccine induced a Th1-biased immune response in both BALB/c and C57BL/6J mice may alleviate the concerns associated with vaccine-induced immunopathology that has been raised for SARS and MERS vaccine candidates. Such immunopathology was characterized by a Th2-skewed immune response and eosinophilia and was reported for different vaccines developed for MERS-CoV and SARS-CoV after viral challenge (Weingartl et al., 2004; Czub et al., 2005; Yang et al., 2005; Deming et al., 2006; Yasui et al., 2008; Bolles et al., 2011; Jaume et al., 2012; Tseng et al., 2012; Iwata-Yoshikawa et al., 2014; Honda-Okubo et al., 2015; Agrawal et al., 2016; Hashem et al., 2019).

Taken together, our study confirms the immunogenicity of a VIU-1005 vaccine candidate expressing full-length consensus SARS-CoV-2 S glycoprotein in eliciting strong Th1-skewed and long-lasting protective humoral and cellular immunity. These data strongly support the clinical evaluation of VIU-1005 DNA vaccine as a vaccine candidate against SARS-CoV-2.

## Data Availability Statement

The raw data supporting the conclusions of this article will be made available by the authors, without undue reservation.

## Ethics Statement

The studies involving human participants were reviewed and approved by the Unit of Biomedical Ethics in King Abdulaziz University Hospital (Reference No. 245-20). Signed informed consents were obtained from all participants as per institutional ethical approval. The animal study was reviewed and approved by the Animal Care and Use Committee (ACUC) at King Fahd Medical Research Center (KFMRC), King Abdulaziz University (KAU), Jeddah, Saudi Arabia, and ethical approval was obtained from the Bioethical Committee at King Abdulaziz University (KAU), Jeddah, Saudi Arabia (approval number 04-CEGMR-Bioeth-2020).

## Author Contributions

AA, RYA, MH, and AMH designed and conceptualized the work. SSA, KAA, RYA, MB, AA, SA, MAA, TSA, WHA, M-ZE, RHA, and AMH performed and optimized the experiments and analyzed the data. SSA, KAA, AA, and AMH drafted the manuscript. All authors have reviewed and edited the manuscript and agreed to the published version of the manuscript.

## Conflict of Interest

AMH, MAA, TSA, KAA, SSA, and AA are inventors on a US patent application related to this work. AMH and AA work as scientific consultants in SaudiVax Ltd. and receive fees for consulting. SA and MH are employees of SaudiVax Ltd. and receives salary and benefits. The remaining authors declare that the research was conducted in the absence of any commercial or financial relationships that could be construed as a potential conflict of interest.

## Publisher’s Note

All claims expressed in this article are solely those of the authors and do not necessarily represent those of their affiliated organizations, or those of the publisher, the editors and the reviewers. Any product that may be evaluated in this article, or claim that may be made by its manufacturer, is not guaranteed or endorsed by the publisher.
