# Supplementary material for: Synthetic SARS-CoV-2 Spike-Based DNA Vaccine Elicits Robust and Long-Lasting Th1 Humoral and Cellular Immunity in Mice
Source: Front Microbiol. 2021 Sep 7;12:727455. doi: 10.3389/fmicb.2021.727455 (PMC8454412; doi:10.3389/fmicb.2021.727455)
Supplement: Supplementary file 1 [file Data_Sheet_1.PDF]

Supplementary Materials

Synthetic SARS-CoV-2 spike-based DNA vaccine elicits robust and long-lasting Th1 humoral and cellular immunity in mice

Sawsan S Alamri[1,2], Khalid A Alluhaybi[1,3], Rowa Y Alhabbab[1,4], Mohammad Basabrain[1], Abdullah Algaissi[5,6], Sarah Almahboub[1], Mohamed A Alfaleh[1,3], Turki S Abujamel[1,4], Wesam Abdulaal[2], M-Zaki ElAssouli[1], Rahaf Alharbi[1], Mazen Hassanain[7], Anwar M Hashem[1,8]

[1] Vaccines and Immunotherapy Unit, King Fahd Medical Research Center, King Abdulaziz University, Jeddah, Saudi Arabia

[2] Department of Biochemistry, Faculty of Science, King Abdulaziz University, Jeddah, Saudi Arabia

[3] Faculty of Pharmacy, King Abdulaziz University, Jeddah, Saudi Arabia

[4] Department of Medical Laboratory Technology, Faculty of Applied Medical Sciences, King Abdulaziz University, Jeddah, Saudi Arabia

[5] Department of Medical Laboratories Technology, College of Applied Medical Sciences, Jazan University, Jazan, Saudi Arabia

[6] Medical Research Center, Jazan University, Jazan, Saudi Arabia

[7] Department of Surgery, Faculty of Medicine, King Saud University, Riyadh, Saudi Arabia

[8] Department of Medical Microbiology and Parasitology, Faculty of Medicine, King Abdulaziz University, Jeddah, Saudi Arabia

*** Correspondence:**
Anwar M Hashem
amhashem@kau.edu.sa

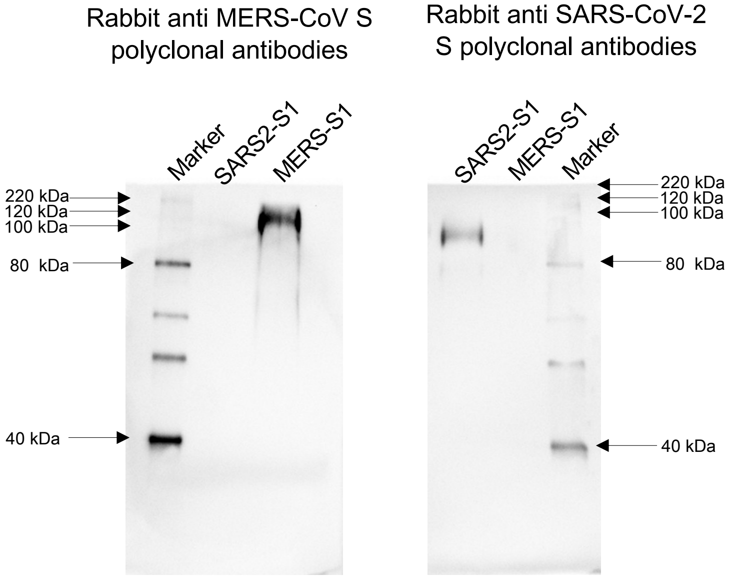

Figure S1. Specificity of the in-house rabbit anti-S (SARS-CoV-2) and (MERS-CoV) polyclonal antibodies. Two NZW rabbits were immunized subcutaneously with recombinant SARS-CoV-2 S protein or MERS-CoV S protein (Sino biological, China) mixed with Freund's Complete Adjuvant at 100 µg per injection, and boosted twice with the same dose every two weeks in Freund's Incomplete Adjuvant. Serum was collected and Western blot confirmed specific binding of each polyclonal antibodies to their respective S1 subunit proteins form SARS-CoV-2 or MERS-CoV but not the other. All animal experiments were conducted in accordance with the Institutional Guidelines and Protocols for Animal Experiments.

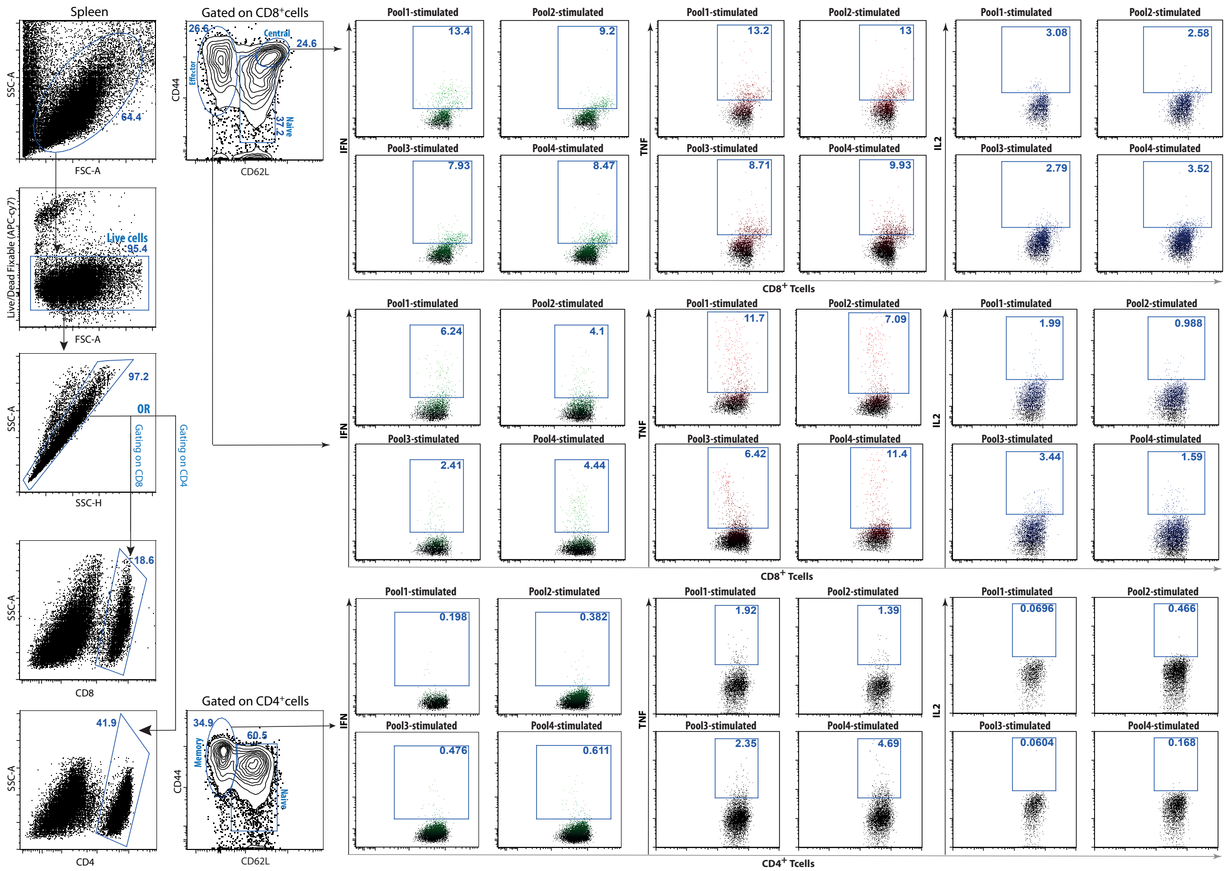

Figure S2. Long-lasting cellular immune response against SARS-CoV-2 S protein in BALB/c mice. Intramuscularly immunized BALB/c mice with 100 µg of VIU-1005 or control plasmid using needle injection were sacrificed at 21 weeks after the last immunization, and splenocytes (n = 3) were isolated and restimulated *ex vivo* with synthetic peptide pools covering SARS-CoV-2 S protein. Figure shows the gating strategy on live CD8$^+$CD44$^+$CD62L$^+$ central memory T cells (CD8$^+$ TCM), effector CD8$^+$CD44$^+$CD62L$^-$ memory T cells (CD8$^+$ TEM), and memory CD4$^+$CD44$^+$CD62L$^-$ T cells. FACS plots display IFN-γ, TNF-α and IL-2 expression on stimulated CD8$^+$ TCM, CD8$^+$ TEM and memory CD4$^+$ T populations from immunized BALB/c mice after restimulation with the different peptide pools. Representative plots are shown.

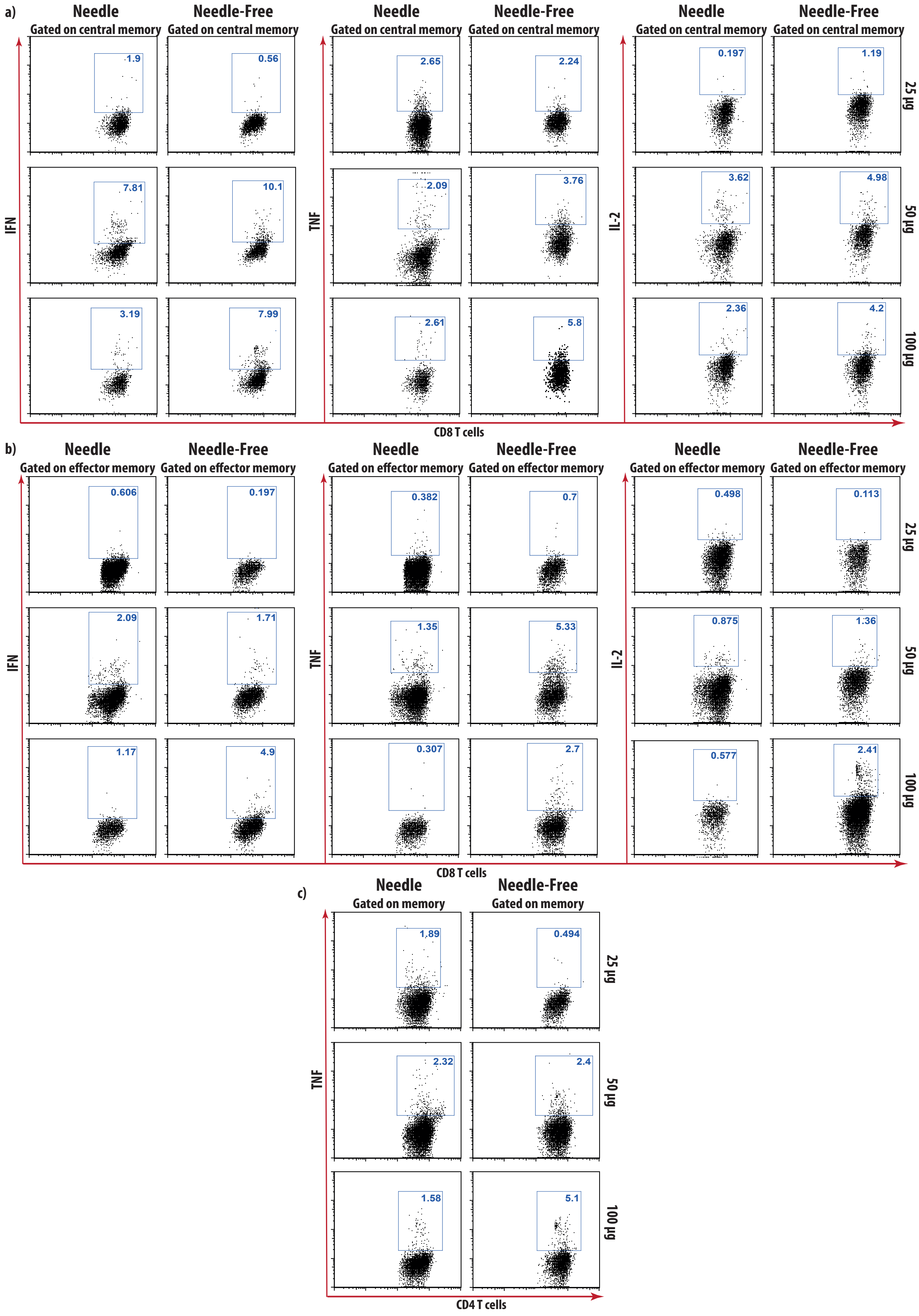
a)
Needle
Gated on central memory
Needle-Free
Gated on central memory
IFN
1.9
0.56
7.81
10.1
3.19
7.99
TNF
2.65
2.24
2.09
3.76
2.61
5.8
IL-2
0.197
1.19
3.62
4.98
2.36
4.2
25 µg
50 µg
100 µg
CD8 T cells
b)
Needle
Gated on effector memory
Needle-Free
Gated on effector memory
IFN
0.606
0.197
2.09
1.71
1.17
4.9
TNF
0.382
0.7
1.35
5.33
0.307
2.7
IL-2
0.498
0.113
0.875
1.36
0.577
2.41
25 µg
50 µg
100 µg
CD8 T cells
c)
Needle
Gated on memory
Needle-Free
Gated on memory
TNF
1.89
0.494
2.32
2.4
1.58
5.1
25 µg
50 µg
100 µg
CD4 T cells

Figure S3. Memory T cell response against SARS-CoV-2 S protein in BALB/c mice intramuscularly immunized with VIU-1005 using either needle-based or needle-free immunization. Intramuscularly immunized BALB/c mice with 3 doses of 25 µg, 50 µg or 100 µg of VIU-1005 plasmid at 2-week intervals using either needle injection or needle-free Tropis system were sacrificed at 8 weeks after the last immunization, and splenocytes (n=3) were isolated and restimulated *ex vivo* with synthetic peptide pool 1 from SARS-CoV-2 S protein. FACS plots show IFN-γ, TNF-α and IL-2 on (**a**) live $CD8^+CD44^+CD62L^+$ central memory T cells ($CD8^+$ TCM) and (**b**) effector $CD8^+CD44^+CD62L^-$ memory T cells ($CD8^+$ TEM). (c) TNF-α expression in memory $CD4^+CD44^+CD62L^-$ T cells. Representative plots are shown.
